# Astrocytes revisited: concise historic outlook on glutamate homeostasis and signaling

**DOI:** 10.3325/cmj.2012.53.518

**Published:** 2012-12

**Authors:** Vladimir Parpura, Alexei Verkhratsky

**Affiliations:** 1 Department of Neurobiology, Center for Glial Biology in Medicine, Atomic Force Microscopy & Nanotechnology Laboratories, Civitan International Research Center, Evelyn F. McKnight Brain Institute, University of Alabama, Birmingham, Ala, USA; 2 Faculty of Life Sciences, The University of Manchester, Manchester, UK; 3 Achucarro Center for Neuroscience, Basque Foundation for Science, Bilbao, Spain; 4 Department of Neurosciences, University of the Basque Country UPV/EHU, Leioa, Spain; 5 Department of Biotechnology, University or Rijeka, Rijeka, Croatia; Parpura and Verkhratsky: Excitable astrocytes

## Abstract

Astroglia is a main type of brain neuroglia, which includes many cell sub-types that differ in their morphology and physiological properties and yet are united by the main function, which is the maintenance of brain homeostasis. Astrocytes employ a variety of mechanisms for communicating with neuronal networks. The communication mediated by neurotransmitter glutamate has received a particular attention. Glutamate is de novo synthesized exclusively in astrocytes; astroglia-derived glutamine is the source of glutamate for neurons. Glutamate is released from both neurons and astroglia through exocytosis, although various other mechanisms may also play a role. Glutamate-activated specific receptors trigger excitatory responses in neurons and astroglia. Here we overview main properties of glutamatergic transmission in neuronal-glial networks and identify some future challenges facing the field.

Astrocytes do not only serve as the metabolic supporting cast for neurons, but are committed to bi-directional signaling with neuronal networks. In the multifaceted interplay between these two principal neural cells, the neurotransmitter glutamate can serve as a common denominator. Metabolically, glutamate and its degradation product glutamine shuttle between astrocytes and neurons in a well described cycle. A key necessity for glutamate-mediated bi-directional heterocellular signaling presents itself in neuronal and astrocytic excitability based on variations of cytosolic Ca^2+^, which is necessary and sufficient to cause exocytotic glutamate release from both cell types. In this article, we first provide a short history of discovery of glial cells in the 19th century, followed by definition of astrocytes and presentation of evolution of these cells in the animal kingdom. We discuss the unique role of astrocytes in the homeostatic control over extracellular concentration of glutamate. Besides being devoted “sponges” removing glutamate from the extracellular space (ECS), astrocytes are the only cells in the brain that synthesize this transmitter de novo. We then re-visit the initial evidence for glutamate-mediated bi-directional signaling between neurons and astrocytes. Before providing the envoi, we put forth an opinion of how the term gliotransmitter should be used as a neurotransmitter that is released by glial cells/astrocytes, but not as a compound solely utilized by these cells. The challenge of sorting out contributions of various mechanism of glutamate release to (patho)physiological conditions in the brain and animal behavior is highlighted. The take home message is that astrocytes play an active role in the mammalian nervous system.

## Early beginnings

The concept of neuroglia as a “substance… which lies between the proper nervous parts, holds them together and gives the whole its form in a greater or lesser degree” was introduced by Rudolf Virchow in 1850s ” [translated from German (1); for more detailed account on the history of glia see (2-4)]. The cellular nature of glial cells was recognized soon after and different types of these cells were morphologically characterized. In 1851, Heinrich Müller identified retinal radial glia, which are known today as Müller cells and were further characterized by Max Schulze in 1858. Also in 1858, Karl Bergmann visualized radial fibers in the cerebellum, which were later identified as glial cells by Camillo Golgi. These cells are generally known as Bergmann glia, although they were also called Golgi epithelial cells. In 1865, Otto Deiters produced the first drawings of stellate glial cells, which most likely were astrocytes. Some years later Jacob Henle and Friedrich Merkel visualized the glial networks in the gray matter.

**Figure 1:**
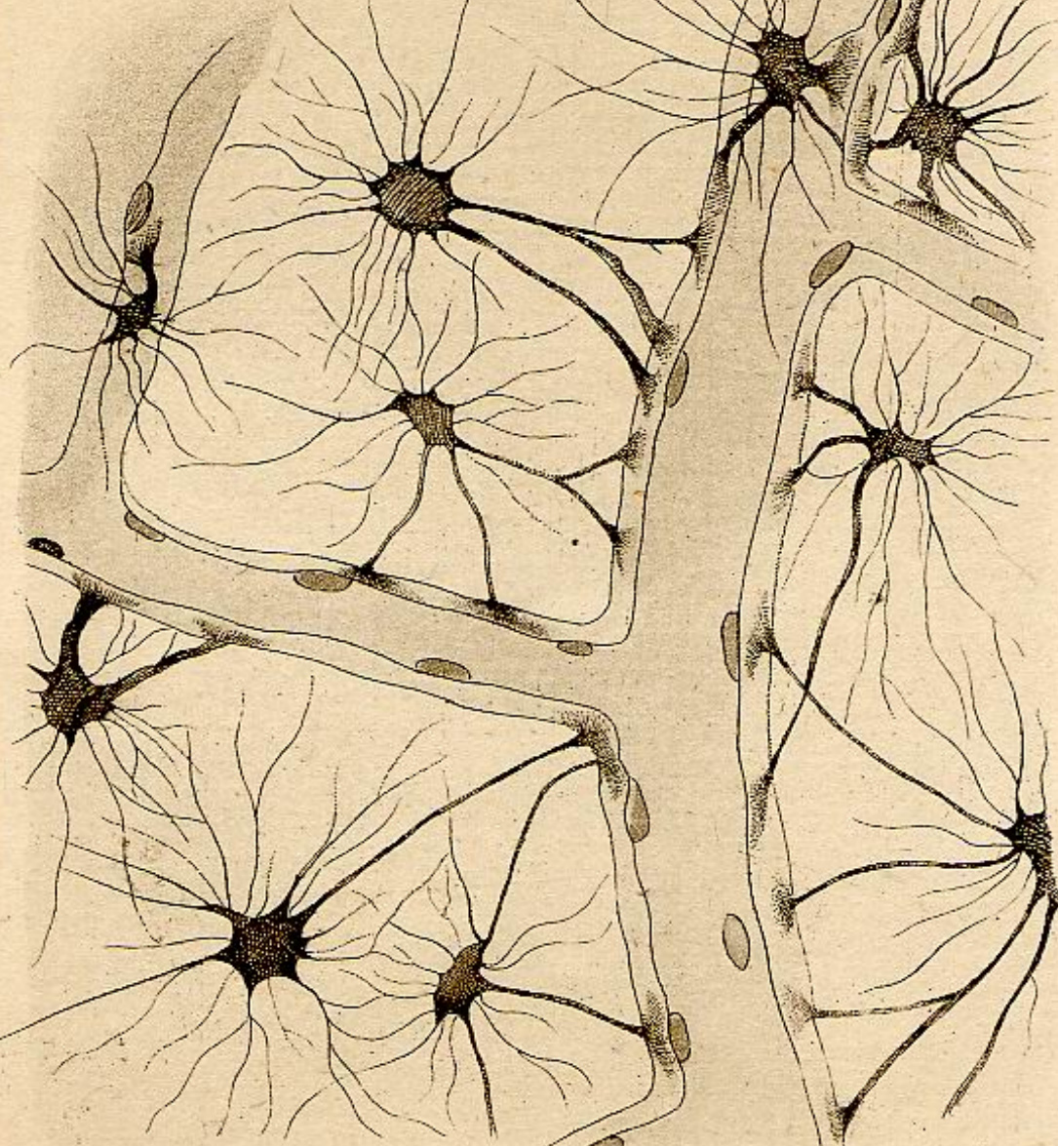
Neuroglial cells drawn by Camillo Golgi. Cells were stained using the silver-chromate technique. Individual star-shaped astrocytes form astroglial network and numerous contacts (the endfeet) with brain capillaries. The image [reproduced from (6)] was kindly provided by Prof. Paolo Mozzarello.

The detailed morphological analysis of glial cells begun after Camillo Golgi, who developed black staining reaction and produced drawings of stained glial cells starting from 1872 (5,6). Golgi described stellate neuroglial cells and found that some glial cells (which were to all probability the protoplasmic astrocytes) send the processes to the blood vessels, where they establish the endfeet structures (Figure 1). He developed a concept of nutritive role of glia and suggested that glial cells establish the metabolic link between blood vessels and the brain parenchyma. In 1889, Wilhelm His made a fundamental discovery of the neural origin of neuroglia by demonstrating that both nerve cells and neuroglia derive from the neuroectoderm (7,8). Soon after, Santiago Ramόn y Cajal developed the first specific stain for astrocytes, the gold chloride-sublimate technique, which, as we know today, labeled glial fibrillary acidic protein (GFAP). Using this technique Cajal confirmed the origin of astrocytes from radial glia, and also demonstrated that astrocytes can divide in the adult brain, thus laying the basis for much later discoveries of the stem properties of astroglia.

## Definition of astrocytes

The name astrocyte (*astron* meaning a star, while *kytos* means a hollow vessel, later a cell; thus, star-like cell) was introduced by Michael von Lenhossek in 1891. Astrocytes are arguably the most numerous and diverse neuroglial cells in the central nervous system (CNS). Surprisingly, there is no clear definition of what astroglial cell is. The general belief that astrocytes are stellate cells that could be distinguished by expression of GFAP does not reflect the reality. Many of astrocytes in the healthy brain do not express GFAP, only some of them have a star-like morphology, and not all of them contact brain vessels. Astrocytes are highly variable in their morphology and function, and astroglial cells in various brain regions may have very different physiological properties. The name astroglia therefore is used as a generic term that could be applied to all those brain cells that are not neurons, oligodendrocytes/NG2 glia, or microglial cells. All these cells, which fall under the above definition of astroglia, have however one thing in common: they are united by their main function that lies in preservation of brain homeostasis. Therefore, astrocytes can be broadly defined as “homeostatic neuroglial cells” critical for sustaining function of healthy brain and fundamental for brain defense in pathology (9-12).

## Evolution of astrocytes

**Figure 2:**
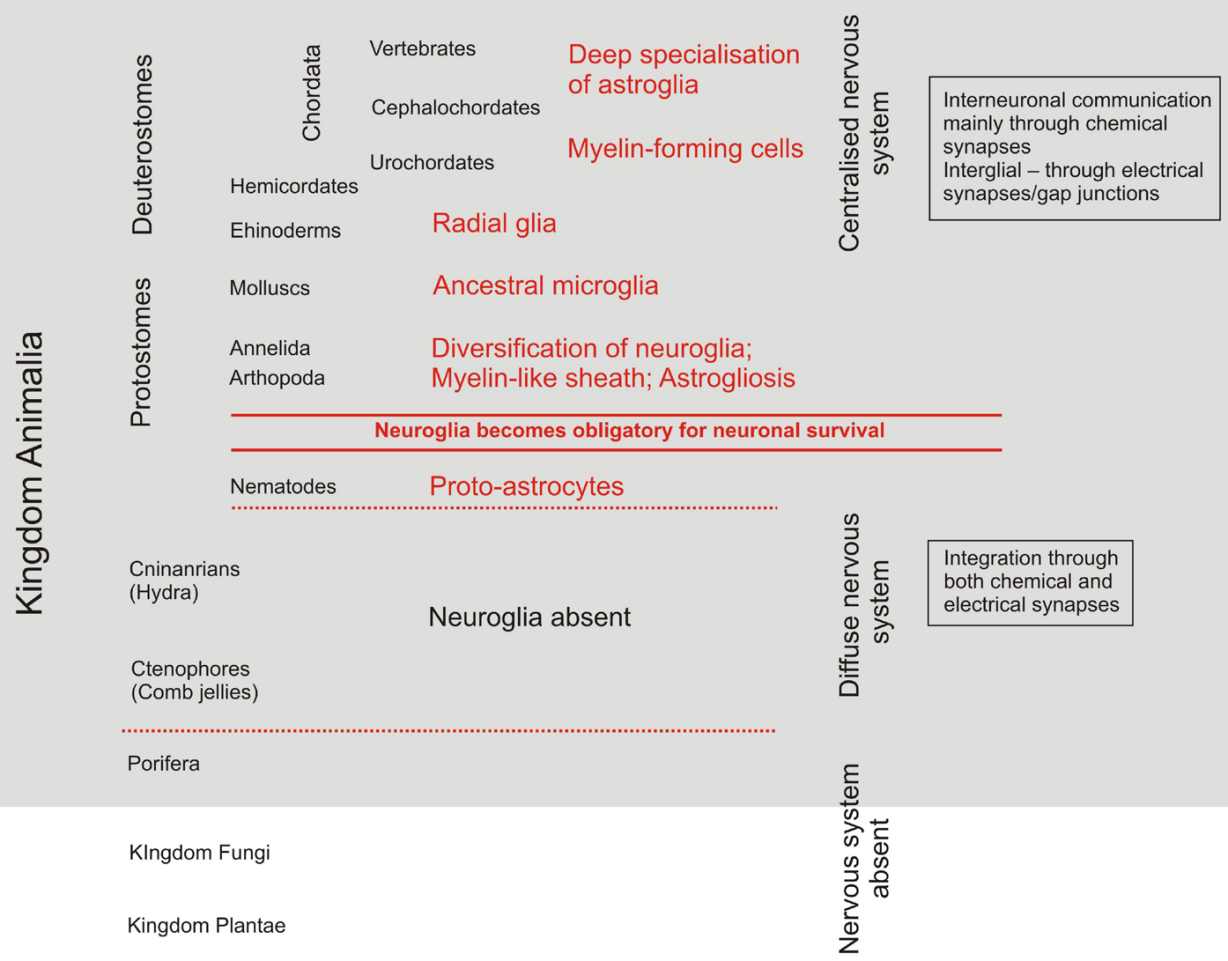
Evolution of neuroglia.

The evolutionary appearance of astrocytes to all probability coincided with the emergence of centralized nervous system (Figure 2) (13). The primordial astrocytes assumed various homeostatic roles, assisting neuronal function and development of the nervous system. In the worm *Caenorhabditis elegans*, 50 proto-astrocytes are associated with the nematode sensory organs or sensilla. Artificial ablation of these proto-astrocytes does not result in neuronal death, but alters neuronal development and affects sensory functions (14). At the higher evolutionary stages, glial cells became much more diverse and much more significant – elimination of astroglia renders nervous system unviable. In Annelides and Arthropodes, multiple types of astroglia control nervous system homeostasis. Astroglial cells also divide neuronal masses into functionally distinct centers. At this evolutionary stage, astrocytes also isolated the nervous system from the rest of the body by forming the blood/hemolymph-brain barrier (BBB/HBB), which appears in crustaceans, insects, and cephalopods. The glial BBB is also found in some vertebrates. For example, in sharks some of the capillaries are completely surrounded by astrocytes, thus, being endocellular vessels. As evolution progressed to higher vertebrates, the BBB function shifted to endothelial cells, which, however, remain under astrocytic control. The ancient astrocytes also ensheath axons, being thus the ancestors of myelin forming oligodendrocytes and Schwann cells. The astroglial coverage of axons found, for example, in earthworms, shrimps and prawns, greatly increases action potential propagation velocity, which in some prawns can approach 200 m/s (15). Finally, astrocytes acquired a defensive function represented by astrogliosis.

## Glutamate homeostasis: metabolic interplay between astrocytes and neurons

An important aspect of astrocytic function in the brain is the homeostatic control over extracellular concentration of glutamate. This performance allows normal operation of synapses and maintains brain parenchyma healthy; prolonged excess of glutamate in the ECS leads to excitotoxicty. Astrocytic function to metabolize and to uptake chemical transmitters was proposed in the first decade of the 20th century by Ernesto Lugaro (16). “Elsewhere I have exposed the arguments that can make us to think that the actions carried out at the level of the neuronal articulations (synapses), between neuronal terminals and dendrites and cellular bodies of subsequent neurons, are of chemical nature. Every nervous ending would undergo in the moment of the excitation a chemical modification and this chemical modification would act as a stimulus on the other neuron. If that were (true), the interneuronal articulations would be a center of active chemical exchanges; and one would therefore comprise the infiltrating of protoplasmatic “tufted” extensions of neuroglia in all the nearby free interstices, in order to perhaps pick and instantaneously to fix even the smallest product of refusal.” [(16); translated from Italian with parenthetical additions for clarification].

Almost a century later, the experimental confirmation of Lugaro’s prophetic words became available. In 1994, Mennerick and Zorumski (17) showed that astrocytes can respond to glutamatergic neurotransmission by activation of the plasma membrane glutamate receptors and transporters. The activation of these transporters in turn affected synaptic transmission, as it contributed to its termination.

**Figure 3:**
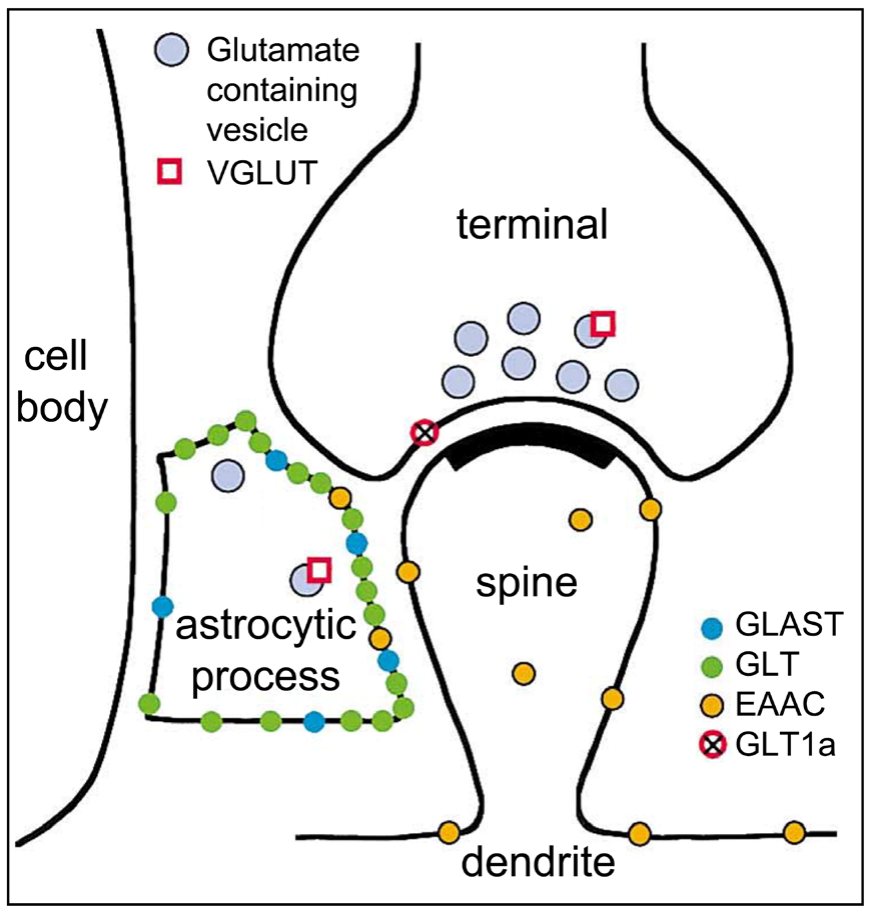
Schematic representation of the localization of glutamate transporters at the hippocampal tripartite synapse. Glutamatergic synapses in the hippocampus are contacted by astrocytes. GLAST and GLT are present in the astrocytic membranes at high density, especially toward the neuropil. Presynaptic terminals specifically contain GLT1a variant. Quantitative information on EAAC is not available. Neurons and astrocytes possess vesicular glutamate transporter (VGLUT)-laden vesicles which contain glutamate. Modified from (18).

The cytoplasmic glutamate concentrations in neurons and astrocytes are differentially maintained and regulated (18,19), which is especially evident at the tripartite synapse (20). Astrocytes remove 80% of glutamate from the ECS, while neurons handle the remaining 20% (18). Glutamate removal is achieved by operation of high affinity glutamate transporters expressed on the plasma membrane (K_m_ ~ 20 μM) (18), localization of which to certain tripartite synapses has been well documented (Figure 3). For instance, while some of glutamatergic synapses in the hippocampus are engulfed by, the others are in partial contact by astrocytes. In the stratum radiatum of hippocampal CA1 region, 57% of the synapses formed between Schaffer collaterals and CA1 pyramidal neurons are bordered by astrocytes (21); astrocytic processes surround somewhat less than half ( ~ 0.4) of the synaptic area and occupy part of the ECS between neighboring synapses. Rodent L-glutamate/L-aspartate transporter (GLAST) and glial L-glutamate transporter 1 (GLT1), highly homologous to human excitatory amino acid transporters (EAAT1 and EAAT2, respectively), are present in the astrocytic membranes at a high density of 2300 and 8500 molecules per μm^2^, respectively, with the highest concentration toward the neuropil (18). Astroglial glutamate transporters are tightly regulated by transmembrane Na^+^ concentration, which couples glutamate transport to astroglial activity; in fact fluctuations in the astroglial Na^+^ control most of homeostatically important transporters (22-25). About 6% of GLT1 can be detected in the plasma membrane of presynaptic terminals, both within and outside of the synaptic cleft (26). It appears that presynaptic terminals are specifically populated by the GLT1a variant (26,27). Although this synapse additionally displays the excitatory amino acid carrier 1 (EAAC), also called EAAT 3; quantitative information on it is not available.

**Figure 4:**
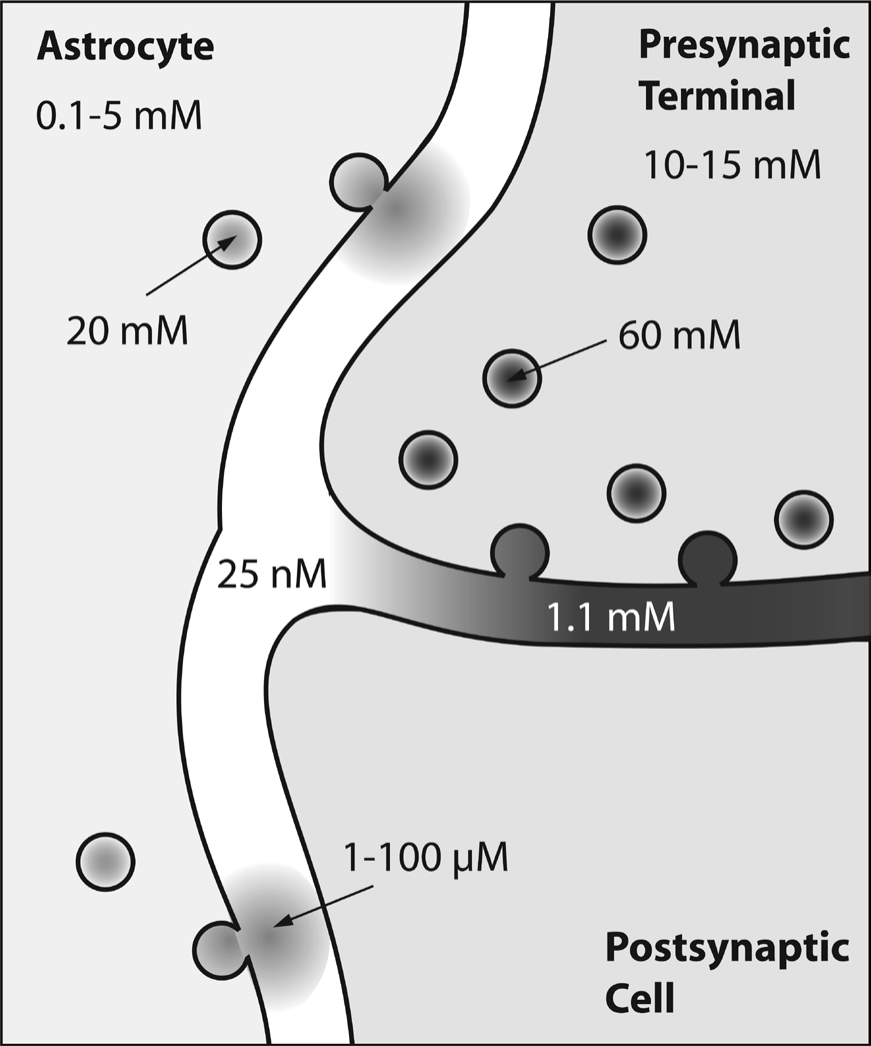
Glutamate concentration in different cellular and extracellular compartments at the tripartite synapse. Modified from (29).

The concerted activity of EAATs maintains extracellular concentration of glutamate low at ~ 25 nM (28) (Figure 4). Once in the cytosol, glutamate is converted to glutamine by the enzyme glutamine synthetase in astrocytes but not in neurons, which do not express this enzyme. In a well described metabolic interplay between neurons and astrocytes, ie, the glutamate-glutamine cycle, glutamine (inert in respect to glutamate receptors) is transported out of astrocytes into the ECS and is taken up by neurons/presynaptic terminals, where it is converted to glutamate via the enzyme glutaminase. In addition, glutamine acts as a primary source for synthesis of γ-aminobutyric acid (GABA), thus being critical for maintenance of inhibitory GABA-ergic transmission. Expression of glutamine synthetase is affected in several types of neurological and neurodegenerative diseases such as for example epilepsy and Alzheimer disease (30-32); this deficit may be linked to compromised synaptic transmission observed in these neurological conditions. In synaptic terminals, glutamate concentration reaches 10-15 mM. In astrocytes, cyotosolic glutamate is estimated to be much lower at 0.1-5 mM, presumably due to glutamate synthetase activity (33) (Figure 4). Since glutamate is also a metabolic fuel which gets oxidatively degraded, it is compensatory net (de novo) synthesized in the brain from glucose. It is essential to realize that glucose is the only glutamate precursor that crosses the BBB in sufficient quantities; needleless to say glutamate does not cross an intact BBB. Astrocytes not only have their endfeet at blood capillaries delivering glucose, but they also (unlike neurons) express the mitochondrial enzyme pyruvate carboxylase. Owing to the entry of pyruvate, which gets generated by glucose degradation in the cytosol, to the tricarboxylic acid cycle via this enzyme, glutamate is net synthesized as a by-product of the cycle (34). Therefore, in astrocytes and neurons the sources of cytoplasmic glutamate are dual. Through the activity of EAATs both cell types take up glutamate from the ECS, which represent one (indirect) source. The other source is differential. In astrocytes, glutamate is derived from de novo synthesis (direct source), while in neurons it is converted from glutamine, provided by astrocytes (indirect source).

Both neurons and astrocytes (albeit to a lesser degree) express various isoforms of the vesicular glutamate transporter (VLGUT) (Figure 3), which mediates packaging of glutamate into secretory vesicles. The intravesicular concentration of glutamate reaches ~ 60 mM in neurons and ~ 20 mM in astrocytes (29) (Figure 4). Ca^2+^-dependent exocytosis at pre-synaptic terminal raises the concentration of glutamate in the synaptic cleft to ~ 1.1 mM (35), while in astrocytes it results in release with localized extracellular glutamate accumulation of 1-100 µM (36) (Figure 4).

## Glutamate signaling between neurons and astrocytes

Astrocytes engage in the bi-directional glutamatergic communication with neurons, in which both cells use vesicular mechanism of glutamate release (37). Rather than making an extensive coverage of literature on this topic available elsewhere (38,39), we shall briefly describe some pivotal studies that were instrumental to establish this astrocytic function. A prerequisite for the existence of receptor-mediated signaling in astrocytes is the expression of glutamate receptors. Indeed, astrocytes possess functional glutamate receptors of both ionotropic (iGluRs) and metabotropic (mGluRs) kinds, the discovery of which dates back to the early 1980s (40-44).

**Figure 5:**
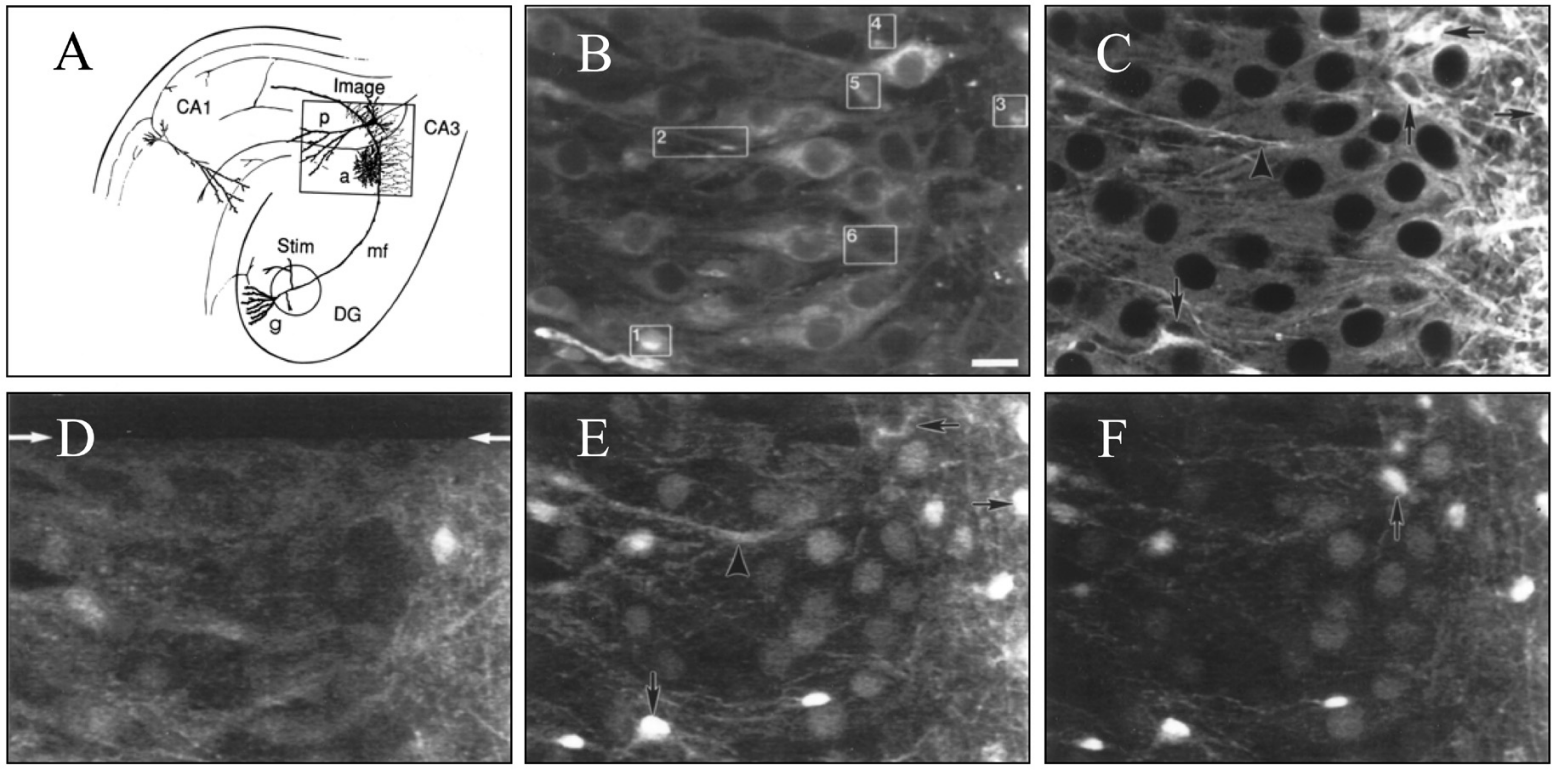
High frequency (50 Hz) electrical stimulation of mossy fibers evokes [Ca^2+^]_i_ responses in CA3 region astrocytes. (**A**) Drawing shows positioning of stimulating electrode (Stim) and imaged area (rectangular area) shown in B-F with respect to microanatomy of transverse hippocampal slice. Stimulating electrode is placed in dentate gyrus (DG) to depolarize axons of granule cells (g), which synapse onto pyramidal neurons (p) in CA3 region. Cell bodies of pyramidal neurons form palisades (dotted outlines) and are surrounded by astrocytes (a). (**B**) Resting fluorescence observed within CA3 region after loading slices with Ca^2+^ indicator fluo-3 (32 frame average). Note the stratum pyramidale (palisades) formed by neuronal somata (left 2/3 of the image), while stratum lucidum lies to the far right. The squares numbered 1-5 indicate astrocytes, while 6 indicates a neuron.(**C**) GFAP immunofluorescent reactivity of the field shown in B acquired after the Ca^2+ ^imaging sequence shown in D-F (single frames). Arrows and arrowhead indicate astrocytic somata and processes, respectively. (**D**) The earliest response in CA3 region following electrical stimulation of dentate gyrus. The arrows indicate the active horizontal scan line at the time of stimulus onset (*t* = 0 seconds). Thus, the portion of the image above the line represents fluorescence before stimulation, while the portion of the image below the line reports on fluorescence intensities after electrical stimulation, showing [Ca2+]_i_ increases in pyramidal cell bodies and fine neuronal processes. (**E**) After an additional 2 seconds of stimulation (t = 4 seconds), the pyramidal cell bodies exhibited large [Ca2+]_i_ increases, but also many GFAP-positive cell bodies and processes showed [Ca2+]_i_ increases. Arrows and arrowhead in E and F correspond to those in C. (**F**) After 4 seconds of stimulation (t = 6 seconds) almost all astrocytes within the imaging area responded with [Ca2+]_i_ increases. Scale bar, 20 μm. Modified from (46). Data courtesy of Dr Stephen J. Smith, Stanford University.

Glutamate, by activating specific receptors, triggers cytosolic Ca^2+^ increases in astrocytes, as has been initially demonstrated in cultured hippocampal astrocytes (45). This seminal finding opened a possibility that astrocytes could receive glutamatergic signals from neurons during synaptic transmission. Such glutamate-mediated neuron-astrocyte signaling was experimentally characterized in organotypic slice cultures of rat hippocampus (46). Mossy fibers originating in the dentate gyrus of the hippocampus and projecting onto CA3 pyramidal neurons were electrically stimulated, while monitoring intracellular Ca^2+^ levels ([Ca^2+^]_i_) in neurons and astrocytes of the CA3 region (Figure 5). Identification of astrocytes in slices was established by post-recording GFAP immunocytochemistry. Using stimulation (8 Hz) of mossy fibers, the authors observed [Ca^2+^]_i_ increases in both neurons and astrocytes within the CA3 area. Neuronal [Ca^2+^]_i_ reached steady state levels within several seconds, while the astrocytic population, located in stratum radiatum, lucidum, and oriens, and also intermingled among pyramidal cell somata, started to show substantial increases in [Ca^2+^]_i_ after several seconds of mossy fiber stimulation. The delay in the astrocytic response was shortened, to within a 2-second minimal latency, when a higher frequency (50 Hz) stimulus was used. All astrocytic [Ca^2+^]_i_ responses to neuronal stimulation were blocked by kynurenic acid, a broad-spectrum GluR antagonist. Thus, astrocytes in hippocampal organotypic slice cultures respond to the glutamate released from neurons. This glutamate-mediated neuron-astrocyte signaling pathway was subsequently confirmed in acute hippcampal (47) and ventrobasal thalamic (48) slices.

Secretion from astrocytes was proposed in the early 20th century by Hans Held (49) and Jean Nageotte (50,51). Held discovered granular inclusions in processes of specialized astrocytes, marginal (subpial) glial cells. These granular inclusions were referred to as “gliosomes” by Alzheimer (52), and observed by many microanatomists of that time (the year of reporting in parentheses): Eisath (1906), Fieandt (1910), Cajal (1913), Achucarro (1913), Hortega (1916), and Penfield (1924) (51). However, this original term must not be confused with the recent reincarnation of it (53) for glial subcellular re-sealed particles (54), which represent secretion competent pinched off astrocytic processes that contain vesicles filled with transmitter(s) (53). Nonetheless, Held speculated that gliosomes represent evidence for glial secretion, while the first description of secretion in neuroglia came from Nageotte.

**Figure 6:**
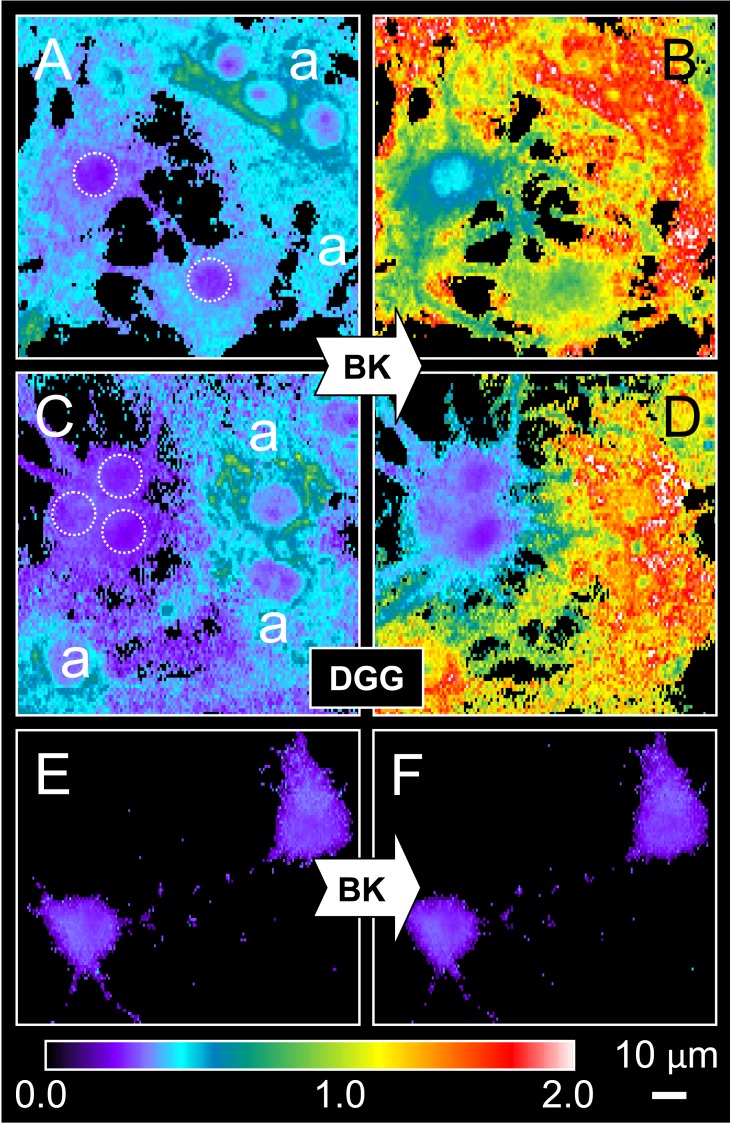
Bradykinin causes a glutamate-mediated accumulation of internal Ca ^2+^ in neurons co-cultured with astrocytes. The [Ca^2+^]_i_ in neocortical neurons (dotted circles in A and C) and astrocytes (a in A and C) was monitored using fura-2. (**A**) Mixed culture at rest. (**B**) Application of bradykinin (BK, 1 μM, 75 seconds) caused an elevation in [Ca^2+^]_i_ in astrocytes and neurons. (**C**) However, when co-cultures were bathed in presence of the broad spectrum GluR antagonist D-glutamylglycine (DGG), application of bradykinin did not significantly alter neuronal [Ca^2+^]_i_ calcium levels, even though bradykinin elevated the astrocytic [Ca^2+^]_i_ (**D**). (**E-F**) Bradykinin failed to elevate [Ca^2+^]_i_ in neurons devoid of surrounding astrocytes. Color scale indicates pseudocolor representation of [Ca^2+^]_i_, by fura-2 emission ratio ranging from 0 to 2.0. Modified from (55).

The finding that astrocytes can use their Ca^2+^ excitability to release glutamate, which in turn can signal to adjacent neurons came from initial experiment in cortical cell culture (55). Three different means to stimulate cultured astrocytes from visual cortex were used: neuroligand bradykinin and mechanical and photo stimulations. Each of these stimuli raised [Ca^2+^]_i_ in astrocytes and caused glutamate-dependent elevation of [Ca^2+^]_i_ in neighboring neurons. Since similar results were obtained for all three stimuli, the present discussion focuses on experiments using bradykinin. This nonapeptide caused astrocytic [Ca^2+^]_i_ elevations, which were necessary and sufficient to cause glutamate release from these cells; such Ca^2+^ dependency pointed to exocytosis as underlying mechanism of release. Indeed, this novel release mechanism in astrocytes was later confirmed as regulated exocytosis utilizing glutamatergic vesicles (29,56-59). When neurons were co-cultured with astrocytes, application of bradykinin caused an increase in astrocytic and neuronal [Ca^2+^]_i_ (Figure 6 A-B). Broad spectrum GluR antagonist, D-glutamylglycine prevented bradykinin-induced Ca^2+^ accumulations in neurons, without altering astrocytic Ca^2+^ responses to bradykinin (Figure 6 C-D). Furthermore, bradykinin did not cause increase of [Ca^2+^]_i_ in neurons lacking neighboring astrocytes (Figure 6 E-F). Taken together, these data suggested that bradykinin elevates neuronal Ca^2+^ indirectly via glutamate released from astrocytes in response to bradykinin (55). The existence of glutamate-mediate astrocyte-neuron signaling pathway was subsequently confirmed in hippocampal astrocyte-neuron co-cultures (60-62), and in acute hippocampal (63,64) and ventrobasal thalamic slices (48).

## Challenges

To emphasize the source of a neurotransmitter released from glia the term gliotrasmitter was introduced (65). The initially provided set of qualifying criteria for a substance to act as a gliotransmitter (65) was subsequently and repeatedly modified (66-68). Presently gliotransmitter is a chemical released from glial cells which fulfills a working set of criteria: i) it is synthesized by and/or stored in glia; ii) its regulated release is triggered by physiological and/or pathological stimuli; iii) it causes rapid (milliseconds to seconds) responses in neighboring cells; and iv) it has a role in (patho)physiological processes.

It should be noted that besides astrocytes other glial cells were marginally considered in the above endeavor, so that a gliotransmitter should have been termed an “astrotrasmitter.” However, the introduction and usage of the term “gliotransmitter” (or astrotransmitter) may be misleading as it could be interpreted to delineate glial signaling molecules apart from neuronal ones, which is mainly not the case. For instance, besides glutamate, astroglial cells in different brain region were found to secrete classical neurotransmitters and neuromodulators, such as ATP, GABA, D-serine, taurine, or atrial natriuretic peptide (29,56,69). These substances are not confined to glia, as they are also synthesized/found in and released by neurons. Highly likely, the only neuromodulator that can be specific for astroglia is kynurenic acid (70,71).

The molecules involved in chemical transmission between neural cells (ie, neurons and neuroglia) should, by definition, be called neurotransmitters and neuromodulators, which mediate homocellular signaling (neuron to neuron, or astrocyte to astrocyte) and/or heterocellular signaling (neuron to astrocyte and other cells, or astrocyte to neuron and other cells).

While we focused on exocytotic release of glutamate, this transmitter alone can be released from astrocytes by several additional mechanisms: (i) reversal of uptake by plasma membrane glutamate transporters (72), (ii) anion channel opening induced by cell swelling (73), (iii) glutamate exchange via the cystine-glutamate antiporter (74), (iv) release through ionotropic purinergic receptors (75), and (v) functional unpaired connexons, “hemichannles,” on the cell surface (76). Furthermore, as we allude to above, astrocytes can release a variety of neuroligands, including different amino acids, purines, and peptides (56,69). Thus, it will be necessary to catalog whether the same transmitter and/or its release mechanism(s) that operate under physiological conditions operate during pathological conditions or whether there are specific transmitter(s) and/or release mechanism(s) that operate under particular conditions.

Release of transmitters from astrocytes can modulate synaptic transmission and plasticity, leading to changes in behavior (11). For instance, physiological adenosine-mediated astrocyte-neuron signaling appears to be via exocytosis-dependent ATP release from astrocytes followed by extracellular conversion to adenosine (77). This signaling pathway results in modulation of synaptic plasticity in the hippocampus and it is also essential for the process of sleep homeostasis and for responses to sleep deprivation (78), a finding vindicating 1895 speculation made by Santiago Ramón y Cajal that astrocytes control sleep and waking.

Release of transmitters from astrocytes is also involved in pathophysiological behavior. A reduction in extracellular glutamate by withdrawal from cocaine is attributed to compromised cystine-glutamate antiporter (x_c_- system) function (79). Behavioral studies with rats (80) showed that restoring extracellular glutamate with systemic administration of cysteine prodrugs prevented the reinstatement of cocaine seeking. This effect was reversed by the application of a specific mGluR 2/3 antagonist, indicating that glutamate released via the x_c_-system activates inhibitory presynaptic mGluR2/3 receptors, which reduced synaptic glutamate release, and thus prevents drug seeking.

Identifying genes and their products that are responsible for mediating the astrocytic transmitter release and consequential animal behavior will be necessary. The novel genetic approaches using astrocyte-specific conditional expression (77) or deletion (81) of gene products hold promise for assessing the contribution of various transmitter release pathways in astrocytes in physiology and pathology in vivo*.* Certainly, development of additional research tools will be needed to unveil the full extent of astroglial contribution to the brain functions.

## Envoi

The intent of this treatise was to concisely present a historic outlook on glutamate homeostasis and signaling from the perspective of astocytic functions in the brain. If history be our guide, we have just only started to understand the complexity and diversity of population of cells that we termed astrocytes.
